# The Gut Microbiome in Neuromyelitis Optica

**DOI:** 10.1007/s13311-017-0594-z

**Published:** 2017-12-26

**Authors:** Scott S. Zamvil, Collin M. Spencer, Sergio E. Baranzini, Bruce A. C. Cree

**Affiliations:** 1 Department of Neurology, University of California, San Francisco, CA USA; 2 Program in Immunology, University of California, San Francisco, CA USA

**Keywords:** Neuromyelitis optica, AQP4, Microbiome, T cells, Molecular mimicry

## Abstract

Neuromyelitis optica (NMO) is a rare, disabling, sometimes fatal central nervous system inflammatory demyelinating disease that is associated with antibodies (“NMO IgG”) that target the water channel protein aquaporin-4 (AQP4) expressed on astrocytes. There is considerable interest in identifying environmental triggers that may elicit production of NMO IgG by AQP4-reactive B cells. Although NMO is considered principally a humoral autoimmune disease, antibodies of NMO IgG are IgG1, a T-cell-dependent immunoglobulin subclass, indicating that AQP4-reactive T cells have a pivotal role in NMO pathogenesis. When AQP4-specific proliferative T cells were first identified in patients with NMO it was discovered that T cells recognizing the dominant AQP4 T-cell epitope exhibited a T helper 17 (Th17) phenotype and displayed cross-reactivity to a homologous peptide sequence within a protein of *Clostridium perfringens*, a commensal bacterium found in human gut flora. The initial analysis of gut microbiota in NMO demonstrated that, in comparison to healthy controls (HC) and patients with multiple sclerosis, the microbiome of NMO is distinct. Remarkably, *C. perfringens* was the second most significantly enriched taxon in NMO, and among bacteria identified at the species level, *C. perfringens* was the one most highly associated with NMO. Those discoveries, along with evidence that certain Clostridia in the gut can regulate the balance between regulatory T cells and Th17 cells, indicate that gut microbiota, and possibly *C. perfringens* itself, could participate in NMO pathogenesis. Collectively, the evidence linking microbiota to humoral and cellular immunity in NMO underscores the importance for further investigating this relationship.

**Electronic supplementary material:**

The online version of this article (10.1007/s13311-017-0594-z) contains supplementary material, which is available to authorized users.

## Introduction

Neuromyelitis optica (NMO) is a rare central nervous system (CNS) autoimmune inflammatory demyelinating disease characterized by attacks of transverse myelitis and optic neuritis that may lead to severe, disabling paralysis and loss of vision [1]. For many years, NMO was thought to be a severe atypical form of multiple sclerosis (MS), a more common CNS inflammatory demyelinating disease that is considered to target oligodendrocyte-derived myelin proteins [2]. In contrast with MS, distinguished by disseminated white matter lesions containing predominantly lymphocytes, NMO lesions are restricted primarily to the spinal cord, brainstem and optic nerves, and are characterized by the presence of neutrophils and eosinophils, and deposition of antibody and complement [3]. Understanding of the pathogenesis of NMO has advanced rapidly since 2004, when it was discovered that NMO is associated with the serologic biomarker, NMO IgG [4]. Shortly thereafter, aquaporin-4 (AQP4), a water channel protein expressed abundantly on astrocyte end-foot membranes in areas contacting the blood–brain barrier (BBB) [5], was identified as the primary target of NMO IgG [6]. The antibodies of NMO IgG target conformational determinants exposed on extracellular loops of AQP4. Experimental evidence has confirmed that those AQP4-specific antibodies, together with complement, can injure or destroy astrocytes [7]. NMO is therefore considered primarily an autoimmune astrocytopathy.

Despite identification of AQP4 as the principal target, the initial pathophysiologic events that lead to development of NMO have remained elusive. Besides genetic factors that may predispose to NMO, investigators have considered possible environmental triggers, including plants, bacteria, or viruses that could elicit AQP4-specific antibodies [8–10]. “Molecular mimicry”, which can occur when a foreign protein that shares structural or amino-acid sequence homology with a self-antigen elicits cross-reactive immunity [11], is implicated in the pathogenesis of several rheumatologic and CNS autoimmune disorders [12–15]. In this context, some researchers have focused on the identification of foreign proteins that share structural or amino-acid sequence homology with AQP4 [9, 10]. One search of bacterial and viral proteins revealed extensive homology between a *Klebsiella pneumoniae* transmembrane protein and AQP4 but found no evidence for cross-reactivity in NMO [16]. Other investigators suggested that closely related bacterial aquaporins (e.g., aquaporin-Z [17]) could elicit cross-reactivity and provided some experimental evidence supporting their hypothesis. While much effort has been devoted to understanding the origin and pathophysiologic role of NMO IgG, the potential role of T cells, and cellular immune response in general in AQP4 immunity has received less attention. The AQP4-specific T cell may be the cryptic immunologic linchpin in NMO, providing a link between microbiota and NMO pathogenesis.

## Identification of AQP4-Specific T Cells Suggests a Potential Role for Commensal Gut Bacteria in NMO Pathogenesis

Several early observations suggested that T cells participate in NMO pathogenesis. First, the AQP4-specific antibodies of NMO IgG are IgG1, a T-cell-dependent immunoglobulin subtype. Some data suggest that T follicular helper cells, the CD4^+^ T-cell subset that directs B-cell maturation, isotype switching, and differentiation to Ig-secreting plasma cells [18], are elevated in NMO [19, 20]. Second, epidemiologic and genetic studies most frequently associate NMO occurrence with certain allelic major histocompatibility complex (MHC) class II genes, which encode the transmembrane proteins expressed on antigen presenting cells (APCs) that associate with peptide fragments and are presented to antigen-specific CD4^+^ T cells. In this regard, several NMO studies have identified over-representation of patients carrying HLA-DR1*0301 (DR17), DRB3*0202, and DPB1*0501 genes in different ethnic populations [21–23]. Furthermore, HLA-DRB1*1501, the most common MS susceptibility allele, is not associated with NMO [24]. Third, despite the predominance of neutrophils and eosinophils, T cells are also detected in NMO lesions [3, 25], and elevated levels of interleukin (IL)-17 and interferon-γ (proinflammatory T-cell-derived cytokines) have been detected in the cerebrospinal fluid of patients with NMO [26, 27]. Thus, besides directing antibody production by AQP4-reactive B cells, T cells likely contribute to the development of NMO lesions. In this respect, in 2009 it was observed that neither recombinant AQP4-specific antibodies [28] nor NMO IgG alone [29] were pathogenic *in vivo*. However, those AQP4-specific antibodies produced NMO-like lesions in rats that had received encephalitogenic myelin-specific T cells, findings that are consistent with the notion that cellular immune-mediated CNS inflammation causing loss of integrity of the BBB may be a prerequisite for CNS penetration of AQP4-specific antibodies. Collectively, these observations inspired researchers to search for AQP4-specific CD4^+^ T cells in NMO.

**Fig. 1:**
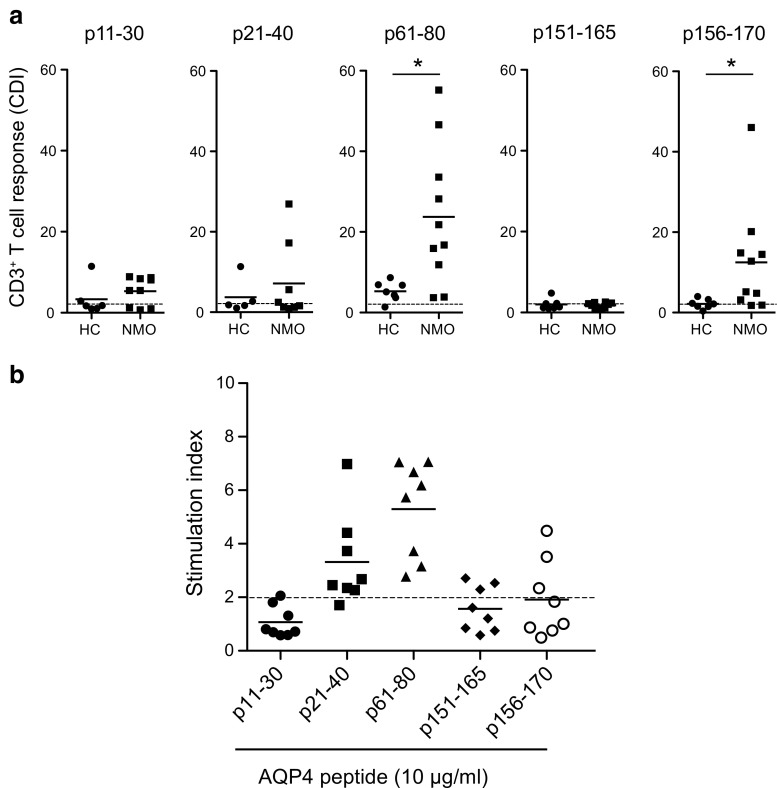
CD4^+^ T cells from patients with neuromyelitis optica (NMO) recognize discrete aquaporin-4 (AQP4) epitopes. (A) Peripheral blood mononuclear cells (PBMC) from patients with NMO and healthy controls (HC) were labeled with 5,6-carboxyfluorescein diacetate succinimidyl ester (CFSE) and stimulated with AQP4 peptides. Proliferation of CD4^+^ T cells was measured by CFSE dilution. Cell division index (CDI) > 2 (broken lines) was considered positive. (B) Recall T-cell proliferation to individual AQP4 peptides was detected by [^3^H]-thymidine incorporation after initial stimulation with recombinant human AQP4. Adapted from Varrin-Doyer, et al., Ann Neurol 72:53-64 (2012). Reproduced with permission of John Wiley & Sons [31]

Working around the same time, investigators from Japan [30], the USA [31], and Israel [32] identified AQP4-reactive T cells in patients with NMO. All 3 groups observed that the numbers of AQP4-reactive T cells were elevated in NMO in comparison with HC. The 2 teams that studied T-cell responses to overlapping 20-mer peptides (p) spanning the length of AQP4 identified multiple determinants, which included an epitope within p61-80 [30, 31]. When our group measured T-cell activation and proliferation, p61-80 was identified as the one AQP4 determinant recognized most frequently in patients with NMO [31] (see Fig. 1). CD4^+^ T cells from patients with NMO that recognized this immunodominant AQP4 T-cell determinant exhibited T helper (Th)17 polarization [31], an observation that added key support to the existing clinical and histologic evidence indicating that Th17 cells have a central role in NMO pathogenesis.

In general, CD4^+^ T cells recognize linear peptide fragments of 12 to 14 amino acids that are produced during protein degradation by APCs [33]. By examining the fine specificity of T cells targeting the immunodominant AQP4 p61-80, its epitope was mapped to amino-acid residues 63 to 76. A search for homologous proteins revealed that this epitope contained a 10-residue sequence (66–75) with 90% homology to a sequence (207–216) within the adenosine triphosphate binding cassette transporter permease (ABC-TP) expressed by *Clostridium perfringens*, a ubiquitous anaerobic Gram-positive spore forming bacteria found in human commensal gut flora. Such a high level of homology is nearly unprecedented. In comparison, studies of T-cell molecular mimicry in MS have identified and focused on T-cell epitopes of myelin antigens that frequently share much less homology with foreign antigens [34–36]. We observed that Th17 cells from patients with NMO, which recognized the immunodominant AQP4 epitope also proliferated in response to the corresponding *C. perfringens* ABC-TP peptide. Our serendipitous discovery suggesting a connection between *C. perfringens* and AQP4-specific T-cell reactivity in NMO could not be overlooked. Together with the emerging appreciation that gut microbiota can influence cellular and humoral immunity, these observations provided a clear foundation justifying the examination of gut microbiota in NMO.

## Analysis of NMO Gut Microbiota Reveals Dysbiosis and Overabundance of *Clostridium perfringens*

Understanding the role of microbiota in human disease is increasing at a tremendous pace [37, 38]. Newborns become colonized with numerous microbes shortly after birth and, while the number of organisms stabilizes after the first year, microbial composition continues to vary in response to environmental changes [39]. Microbiota within the gastrointestinal tract, comprising a community of 10^13–14^ organisms, are particularly heterogeneous [37]. Gram-negative Bacteroides and Gram-positive Firmicutes, including Clostridiales and Lactobacillales, are the major phyla represented in the gut of healthy individuals. It has long been recognized that bacterial species within commensal gastrointestinal flora participate cooperatively in host functions. Specifically, gut microbiota generate metabolites *de novo* and also modify host-derived metabolites, producing certain vitamins, fatty acids, amino acids, and polyamines that are essential to immune regulation or mucosal defense [40, 41]. More recently, shifts within microbial communities have been associated with specific diseases. *Helicobacter pylori* dominates gastric microbiota in peptic ulcer disease, and over-representation of distinct species of gastrointestinal bacteria have been identified in colorectal cancer, type I diabetes mellitus, inflammatory bowel disease, rheumatoid arthritis, Parkinson’s disease, and MS [38, 42–46]. In 2008, it was observed that polysaccharide A (PSA) produced by *Bacteroides fragilis*, which are found within the terminal ileum, promoted expansion of IL-10-producing regulatory T cells (Treg) [47, 48] and a later study showed that PSA expression conferred resistance to experimental autoimmune encephalomyelitis (EAE) in mice [48]. At the time we proposed studying the gut microbiome in NMO, it had been shown that commensal anaerobic spore-forming (chloroform-resistant) *Clostridium* within clusters IV and XIVa, abundant in the colon of mice [49], and that *Clostridia* strains within clusters IV, XIVa, and XVIII isolated from human fecal material [50], also induced Tregs [49, 50]. In contrast, colonization of the terminal ileum with segmented filamentous bacteria, a commensal anaerobic Gram-positive spore-forming bacteria closely related to the genus *Clostridium*, was associated with differentiation of proinflammatory Th17 cells [51] in mice and increased susceptibility to EAE [52]. These findings were particularly intriguing, as differentiation of naive T cells into either Th17 cells or Treg is controlled in a reciprocal manner [53]. Thus, it is plausible that a *Clostridium* species may alter the balance between proinflammatory and anti-inflammatory T-cell subsets in human disease.

**Fig. 2:**
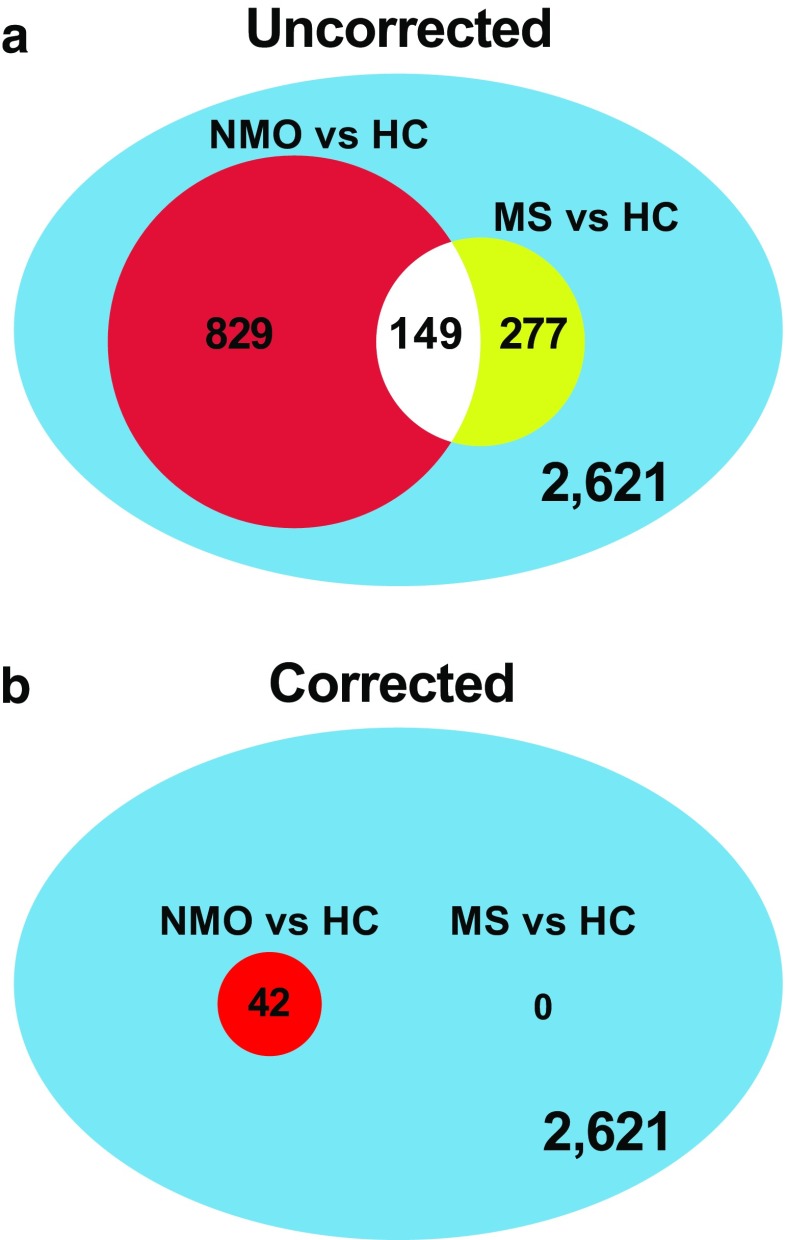
Differential abundance of bacterial taxa in neuromyelitis optica (NMO) and multiple sclerosis (MS) in comparison to healthy controls (HC). A total of 2621 organizational taxonomic units (OTUs) were detected in at least 1 stool sample. (A) Of these, 829 OTUs were differentially abundant between NMO and HC, whereas 277 OTUs were differentially abundant between MS and HC (uncorrected, *p* < 0.05). (B) After correction for multiple comparisons (the threshold for significance is *p* = 1.91 × 10^-5^), 42 OTUs remained differentially abundant for the NMO *vs* HC comparison, whereas no taxa remained associated with MS. Reproduced from Cree, et al., Ann Neurol 80:443-447 (2016), with permission of John Wiley & Sons [54]

**Fig. 3:**
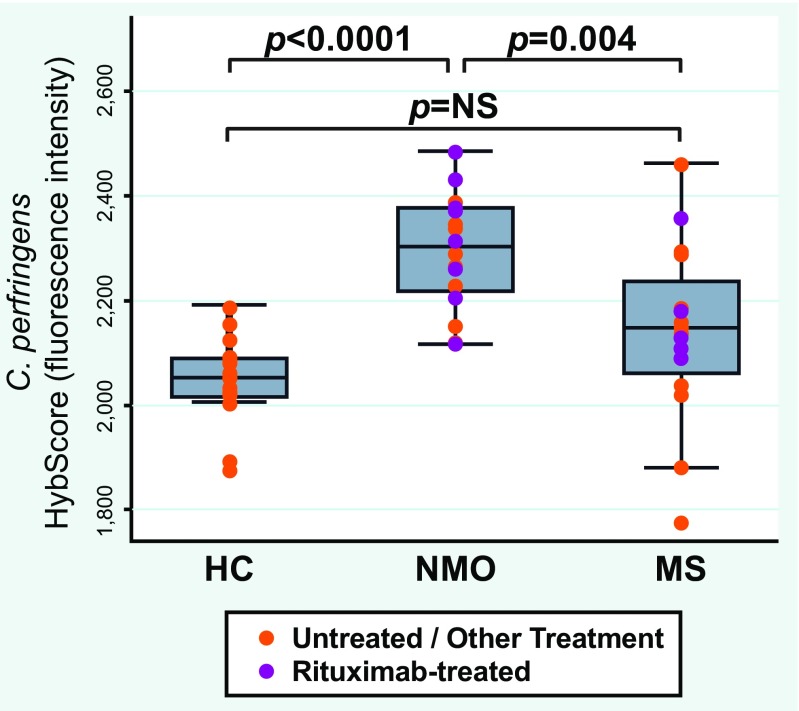
*Clostridium perfringens* abundance was significantly increased in neuromyelitis optica (NMO). The abundance of gut *C. perfringens* was compared between NMO, multiple sclerosis (MS), and healthy controls (HC). Purple dots represent individual values for patients receiving rituximab. The *y*-axis represents the HybScore, a measure of relative abundance. Orange dots represent patients receiving treatment other than rituximab, or no treatment. Reproduced from Cree, et al., Ann Neurol 80:443-447 (2016), with permission of John Wiley & Sons [54]

Currently, the results from 1 NMO gut microbiome study have been reported [54]. In that investigation, we examined stool samples obtained from 16 patients with AQP4-seropositive NMO, 16 patients with MS, and 16 HCs. Principal component analysis demonstrated compositional differences between those bacterial communities. Whereas > 800 organizational taxonomic units were identified that differed in relative abundance between NMO and HC, only 42 retained statistical significance following correction for multiple comparisons (see Fig. 2). In contrast, of nearly 300 organizational taxonomic units that exhibited differential abundance between MS and HCs, none withstood similar correction. Because a majority of the patients with NMO were treated with rituximab or another immunosuppressive treatment, which could hypothetically alter the gut microflora, patients with MS treated with rituximab were included as added controls. Differences in the gut microbiota between rituximab-treated MS patients and rituximab-treated NMO patients were identified, indicating that dissimilarities between NMO and MS could not be attributed entirely to that treatment (see Fig. 3). Thus, regardless of whether the analysis included the entire dataset or individual subsets, results indicated that there are distinct changes in the NMO microbiota.

Remarkably, of bacteria identified at the species level, *C. perfringens* was the species most significantly enriched in patients with NMO compared with HCs (Table 1). Only an unclassified species of Fibrobacteres had a more significant *p*-value. In a 3-way analysis, *C. perfringen*s was overabundant in NMO versus either HC or MS. Although *C. perfringens* was also over-represented in MS samples, the significance was marginal and did not survive statistical correction for multiple comparisons. Rituximab treatment did not influence abundance of *C. perfringens* in patients with either NMO or MS, and *C. perfringens* remained significantly associated with NMO for the comparison of patients with rituximab-treated NMO and rituximab-treated MS.

**Table 1:** Twelve most significant organizational taxonomic units (OTUs) that differentiate neuromyelitis optica (NMO) from healthy controls (HC)*

Phylum	Class	Order	Family	Genus	Species	*p*-value
Fibrobacteres	Unclassified	Unclassified	Unclassified	Unclassified	Unclassified	2.63 × 10^-8^
Firmicutes	Clostridia	Clostridiales	Clostridiaceae	*Clostridium*	*perfringens*	5.24 × 10^–8^
Tenericutes	Mollicutes	Acholeplasmatales	Acholeplasmataceae	*Acholeplasma*	Unclassified	9.21 × 10^–8^
Firmicutes	Clostridia	Clostridiales	Unclassified	Unclassified	Unclassified	2.21 × 10^–7^
Firmicutes	Clostridia	Clostridiales	Lachnospiraceae	*Coprococcus*	Unclassified	2.24 × 10^–7^
Bacteroidetes	Bacteroidia	Bacteroidales	Unclassified	Unclassified	Unclassified	2.68 × 10^–7^
Firmicutes	Clostridia	Clostridiales	Lachnospiraceae	*Blautia*	*producta*	3.95 × 10^–7^
Bacteroidetes	Bacteroidia	Bacteroidales	Prevotellaceae	*Prevotella*	97otu18529	4.71 x 10^-7^
Firmicutes	Clostridia	Clostridiales	Unclassified	Unclassified	Unclassified	6.31 × 10^–7^
Proteobacteria	Alphaproteobacteria	Unclassified	Unclassified	Unclassified	Unclassified	6.75 × 10^–7^
Firmicutes	Clostridia	Clostridiales	Lachnospiraceae	Unclassified	Unclassified	7.33 × 10^–7^
Elusimicrobia	Elusimicrobia	FAC88	91otu12128	94otu9638	97otu81717	7.61 × 10^–7^

*OTUs that significantly differed in abundance between NMO and HC after adjusting for multiple comparisons (*p* < 1.91 × 10^–5^), ranked in order of decreasing statistical significance. Adapted from Cree, et al., Ann Neurol 80:443-447 (2016), with permission of John Wiley & Sons [54]

## Potential Roles of *Clostridium perfringens* in NMO Pathogenesis

**Fig. 4:**
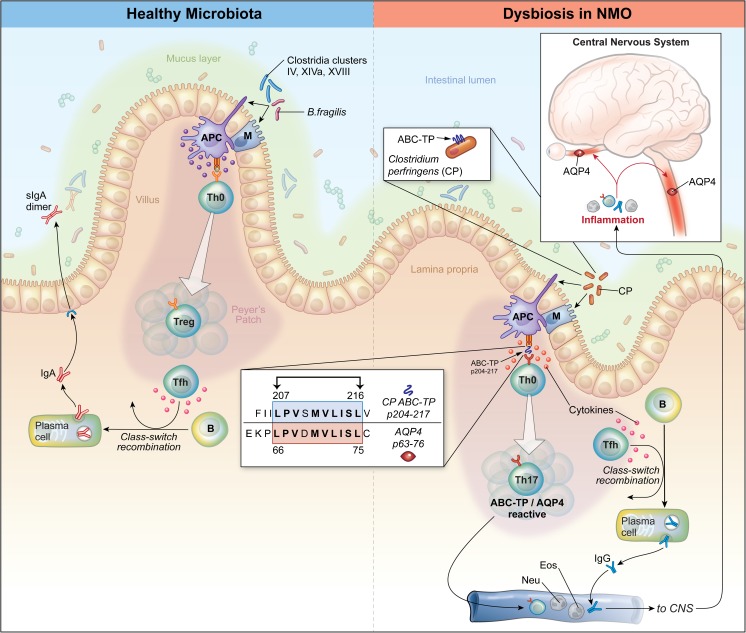
Model illustrating potential roles of *Clostridium perfringens* in neuromyelitis optica (NMO) pathogenesis. **(**Left) “Healthy microbiota”. Commensal bacteria, including *Bacteroides fragilis* and certain species within Clostridia clusters IV, XIVa, and XVIII, promote T-cell immune regulation [47, 49, 50]. Bacteria bind to M cells, which are concentrated in the terminal ileum and appendix in proximity to Peyer’s patches, a gut-associated lymphoid tissue (GALT), and are highly specialized to engulf microbial antigens and deliver them to antigen presenting cells (APCs) [55, 56], including dendritic cells (DC) and macrophages. APCs may also ingest bacterial antigens directly. The APCs, including regulatory CD103^+^ DC, produce anti-inflammatory cytokines (e.g., transforming growth factor-β) that promote expansion of antigen-specific regulatory T cells (Tregs). Those Tregs, along with T follicular helper cells (Tfh), a specialized subset of T cells that directs B-cell maturation, class-switch recombination, and differentiation into immunoglobulin-secreting plasma cells [18], promote production of bacteria-specific IgA [57, 58], which is the most abundant immunoglobulin subclass in the gastrointestinal tract. Individual IgA molecules enter gut epithelial cells and form IgA dimers, which are secreted (sIgA) into the intestinal lumen and mucus layer where they bind their specific bacterial targets. Bacteria-specific sIgA are known to alter microbiota composition and are thought to protect against inflammation and disease [57–59]. (Right) “NMO dysbiosis”. Overabundance of *C. perfringens* (CP) may elicit proinflammatory aquaporin-4 (AQP4)-specific T-cell and B-cell responses that contribute to development of NMO. CP binds to M cells or APC as described above. Processing of CP by APCs exposes a determinant of the ABC-TP (p204-217) that shares homology to AQP4 (p63-76), and when presented by APC, leads to activation and expansion of T cells that recognize either of these antigens (“molecular mimicry”) [31]. CP may expose products that promote secretion of the APC-derived proinflammatory Th17-polarizing cytokines (e.g., interleukin-6) that are increased in patients with NMO [31, 60, 61] leading to expansion of ABC-TP/AQP4-reactive T cells. Those Th17 cells, along with Tfh within GALT or in other secondary lymphoid tissues, promote AQP4-specific B cells to differentiate into plasma cells that secrete pathogenic AQP4-specific IgG1. In conjunction with other leukocytes (e.g., neutrophils and eosinophils), ABC-TP/AQP4-specific Th17 cells and AQP4-specific IgG target AQP4 in the central nervous system (CNS) causing inflammation of the optic nerves and spinal cord. Image courtesy of Xavier Studio

Two NMO investigations, one that evaluated specificity of AQP4-reactive T cells and one that examined the microbiome, both provided findings that could support a role for *C. perfringens* in NMO. Based upon those results, we hypothesized that *C. perfringens* may have dual functions in NMO pathogenesis: it may 1) serve as its own proinflammatory adjuvant, promoting Th17 polarization; and 2) expose a determinant of a *Clostridium* ABC-TP that cross-reacts with AQP4 leading to expansion of AQP4-reactive T cells (see Fig. 4). Such a bold hypothesis should be balanced by some skepticism. *Clostridium* is ubiquitous, but NMO is rare. So, why would there be an association? Several possibilities exist. *C. perfringens* could act in concert with either, or both, environmental and genetic factors that predispose to NMO. Intrinsic molecular characteristics of Clostridia or its metabolic products, independent of antigenicity, are important. Dietary and endogenous lipid composition influences the balance between Treg and Th17 cells [62]. Specifically, short-chain fatty acids, for example, butyrate, propionate, and acetate, produced by anaerobic commensal bacteria in the colon by metabolism of undigested complex carbohydrates, promote development of Treg [40, 41]. In contrast, long-chain fatty acids, including lauric acid, through their influence on the retinoid orphan receptor gamma t, the central regulator of Th17 differentiation [63], promote expansion of Th17 cells [62, 64]. Reconstitution of germ-free mice with Treg-promoting Clostridia strains from clusters IV, XIVa, and XVIII has been associated with increased levels of short-chain fatty acids and transforming growth factor-β1 in the cecum [50]. However, *C. perfringens* is a species within cluster I [65]. Thus, one can speculate that dysbiosis of *C. perfringens* could influence the balance favoring long-chain fatty acids in the gut in humans, and like segmented filamentous bacteria in mice, promote Th17 differentiation [51]. Similarly, *C. perfringens* might cooperate with other local host metabolic factors, including high dietary salt [66] or epithelial serum amyloid A [67, 68], which can act as adjuvants promoting Th17 differentiation [66–68].

Examples of molecular mimicry are well recognized in autoimmune disorders [12–14]. While our data examining T-cell reactivity to AQP4 support molecular mimicry, it is difficult to imagine how this mechanism could apply to all patients with NMO. First, T-cell antigen recognition is “MHC-restricted” by the capability of a peptide to bind specific (allelic) MHC molecules. However, NMO is associated with multiple allelic HLA-D genes. At this time, it is not clear whether each of those allelic MHC II molecules can serve as restricting elements for presentation of the immunodominant AQP4 determinant or the *C. perfringens* ABC-TP mimic. Second, not all patients with NMO are AQP4 seropositive. Currently, T-cell reactivity to AQP4 has been examined in patients with “classic” AQP4-seropositive NMO but not in AQP4-seronegative patients. Thus, it is unknown whether T cells from AQP4-seronegative patients exhibit similar reactivity to the immunodominant AQP4 T-cell epitope or, alternatively, whether other autoantigens serve as targets in NMO. In this context, in 2014, several groups described patients with an opticospinal inflammatory disease resembling NMO that was associated with antibodies to myelin oligodendrocyte glycoprotein (MOG) and referred to this disorder as MOG NMO spectrum disorder (MOG-NMOSD) [69–71]. Whether this “non-classical NMO-like” disorder is truly NMO was first questioned a short time later [72], and has since remained controversial. Unlike AQP4, which is expressed abundantly on astrocytes, MOG is an oligodendrocyte-produced myelin antigen and is a candidate antigen in MS. Nevertheless, the apparent heterogeneity in NMO or NMO-like conditions highlights concern in identifying one pathogenic mechanism for all similarly affected patients.

Ubiquitous in normal gut flora, *C. perfringens* is a diverse species that is recognized as a pathogen in several conditions. Fewer than 5% produce a *C. perfringens* enterotoxin, encoded by the gene *cpe* located in either the bacterial chromosomal or plasmid DNA [73]. *Clostridium perfringens* strains are classified into 5 subtypes (A–E) according to the particular toxin produced [73, 74]. Type A, which can produce the α enterotoxin, is a common cause of food poisoning and is also associated with gas gangrene. A year after we proposed a potential role for commensal *C. perfringens* in NMO, another group of investigators found evidence suggesting that type B *C. perfringens* might contribute to MS pathogenesis [75]. Type B *C. perfringens* produces an ε toxin (ETX) that has tropism for the CNS and can cause disruption of the BBB and injure neurons, astrocytes, and oligodendrocytes [76, 77]. Our microbiome study [54], which included patients with NMO and MS, and other larger investigations dedicated to MS and HC [45, 46, 78, 79], did not identify statistically significant elevations of *C. perfringens* in MS gastrointestinal microbiota. Nonetheless, it remains possible that *C. perfringens* ETX participates in pathogenesis in a subset of patients with MS. Because the *C. perfringens* ETX can cause BBB damage, BBB disruption facilitates CNS entry of AQP4-specific IgG1, and *C. perfringens* is overabundant in NMO, it may be important to search for the presence and potential contribution of pathogenic *C. perfringens* subtypes in gut microbiota in NMO.

## Future Challenges

Results from our analysis of fecal bacteria in NMO indicated that the gastrointestinal microbiota in NMO is distinct from HC and MS, supporting the hypothesis that there may be dysbiosis of *C. perfringens* [54]. However, other candidate bacteria may participate in NMO pathogenesis. In this respect, certain bacteria (e.g., *Prevotella copri* and an unclassified species of Enterobacteriaceae) had greater effect sizes in comparison to HC, but their associations with NMO were less statistically significant than *C. perfringens* [54]. Further, only *Fibrobacteres*, a genus containing two cellulose-degrading bacterial species found in cattle and pigs [80], was more significantly associated with NMO than *C. perfringens*, and its effect size was similar. The biological significance of these associations is unclear and deserves further investigation. It is also important to recognize that the relative abundance of individual bacterial species is not uniform throughout the gastrointestinal tract. Therefore, examination of microbiota in fecal samples may not entirely reflect the relative abundance of individual species within their preferred niches.

Definitive answers regarding the role of microbiota in NMO will require research in both human subjects and animal models. It is important to replicate the initial analysis of gut microbiota with larger sample sizes. Owing to the potential impact of immunosuppressive therapies on gut microbiota, future studies should include larger numbers of patients with untreated NMO, which presents a logistic challenge as therapy is typically initiated shortly after diagnosis. As NMO is a rare disease, this research will likely require a coordinated effort among many centers. Further, while similar studies may clarify associations between individual bacterial populations and NMO, they will not permit determination of causality. Gastrointestinal dysbiosis may promote proinflammatory T-cell polarization and, conversely, cellular and humoral immunity can influence intestinal microbial community composition (see Fig. 4) [57–59]. Evidence supporting a direct role for gut microbiota from disease-affected patients or a distinct candidate bacterial species, like *C. perfringens*, may be established through colonization of germ-free mice with fecal samples from patients or by monocolonization with individual bacterial species. Recently, a clinical model of autoimmune opticospinal inflammatory disease that is initiated by Th17 AQP4-specific T cells was established [81–84]. The development of this new model had eluded investigators until it was learned that AQP4-specific T cells and B cells are regulated more stringently by mechanisms of central (thymic) and peripheral tolerance than myelin-specific T cells that cause EAE [82–84]. By taking advantage of this model and related models it will now be possible to examine how colonization of gut microbiota from patients with NMO or monocolonization with *C. perfringens* may influence expansion of those pathogenic AQP4-specific T cells *in vivo*.

We have only scratched the surface in understanding gut dysbiosis in NMO. Nevertheless, it is important to consider its potential therapeutic implications. Is it possible to alter the gut microbiota, possibly through diet or fecal microbiota transplantation (FMT), in a beneficial manner in patients with NMO? In this regard, fecal transplantation has been effective in > 80% of patients with severe recurrent diarrhea caused by antibiotic-resistant *C. difficile* colitis [85]. It is important to recognize that manipulating factors, for example, microbial composition, which may have participated at a certain step(s) within the pathogenic pathway of a complex chronic inflammatory disease, may or may not necessarily translate therapeutically. However, given the severity of NMO, the potential benefit of FMT and its low risk, testing FMT in NMO should be given serious consideration. Based upon provocative results from studies involving patients with NMO, we have proposed potential mechanisms to explain how dysbiosis may participate in NMO pathogenesis. While they may be correct, partly right, or incorrect, it is clearly important to test these hypotheses, especially as such investigation may fill critical gaps in our understanding of NMO pathogenesis.

## Electronic Supplementary Material

ESM 1 (PDF 1225 kb)

## References

[1] Wingerchuk DM, Lennon VA, Lucchinetti CF, Pittock SJ, Weinshenker BG (2007). The spectrum of neuromyelitis optica. Lancet Neurol.

[2] Sospedra M, Martin R (2005). Immunology of multiple sclerosis. Annu Rev Immunol.

[3] Lucchinetti CF, Mandler RN, McGavern D (2002). A role for humoral mechanisms in the pathogenesis of Devic's neuromyelitis optica. Brain.

[4] Lennon VA, Wingerchuk DM, Kryzer TJ (2004). A serum autoantibody marker of neuromyelitis optica: distinction from multiple sclerosis. Lancet.

[5] Hubbard JA, Hsu MS, Seldin MM, Binder DK. Expression of the astrocyte water channel Aquaporin-4 in the mouse brain. ASN Neuro 2015;7(5). 10.1177/1759091415605486 PMC4623559 26489685

[6] Lennon VA, Kryzer TJ, Pittock SJ, Verkman AS, Hinson SR (2005). IgG marker of optic-spinal multiple sclerosis binds to the aquaporin-4 water channel. J Exp Med.

[7] Zekeridou A, Lennon VA (2015). Aquaporin-4 autoimmunity. Neurol Neuroimmunol Neuroinflamm.

[8] Sellner J, Hemmer B, Muhlau M (2010). The clinical spectrum and immunobiology of parainfectious neuromyelitis optica (Devic) syndromes. J Autoimmun.

[9] Vaishnav RA, Liu R, Chapman J (2013). Aquaporin 4 molecular mimicry and implications for neuromyelitis optica. J Neuroimmunol.

[10] Kountouras J, Deretzi G, Gavalas E (2013). Aquaporin 4, *Helicobacter pylori* and potential implications for neuromyelitis optica. J Neuroimmunol.

[11] Fujinami RS, Oldstone MB (1985). Amino acid homology between the encephalitogenic site of myelin basic protein and virus: mechanism for autoimmunity. Science.

[12] Cunningham MW (2014). Rheumatic fever, autoimmunity, and molecular mimicry: the streptococcal connection. Int Rev Immunol.

[13] van den Berg B, Walgaard C, Drenthen J, Fokke C, Jacobs BC, van Doorn PA (2014). Guillain–Barré syndrome: pathogenesis, diagnosis, treatment and prognosis. Nat Rev Neurol.

[14] Berer K, Krishnamoorthy G (2014). Microbial view of central nervous system autoimmunity. FEBS Lett.

[15] Barros PO, Linhares UC, Teixeira B (2013). High in vitro immune reactivity to *Escherichia coli* in neuromyelitis optica patients is correlated with both neurological disabilities and elevated plasma lipopolysaccharide levels. Hum Immunol.

[16] Jarius S, Wandinger KP, Platzer S, Wildemann B (2011). Homology between *Klebsiella pneumoniae* and human aquaporin-4: no evidence for cross-reactivity in neuromyelitis optica. A study on 114 patients. J Neurol.

[17] Ren Z, Wang Y, Duan T (2012). Cross-immunoreactivity between bacterial aquaporin-Z and human aquaporin-4: potential relevance to neuromyelitis optica. J Immunol.

[18] Crotty S (2011). Follicular helper CD4 T cells (TFH). Annu Rev Immunol.

[19] Li YJ, Zhang F, Qi Y (2015). Association of circulating follicular helper T cells with disease course of NMO spectrum disorders. J Neuroimmunol.

[20] Fan X, Jiang Y, Han J (2016). Circulating memory T follicular helper cells in patients with neuromyelitis optica/neuromyelitis optica spectrum disorders. Mediat Inflamm.

[21] Matsushita T, Matsuoka T, Isobe N (2009). Association of the HLA-DPB1*0501 allele with anti-aquaporin-4 antibody positivity in Japanese patients with idiopathic central nervous system demyelinating disorders. Tissue Antigens.

[22] Brum DG, Barreira AA, dos Santos AC (2010). HLA-DRB association in neuromyelitis optica is different from that observed in multiple sclerosis. Mult Scler.

[23] Deschamps R, Paturel L, Jeannin S (2011). Different HLA class II (DRB1 and DQB1) alleles determine either susceptibility or resistance to NMO and multiple sclerosis among the French Afro-Caribbean population. Mult Scler.

[24] Cree BA, Reich DE, Khan O (2009). Modification of multiple sclerosis phenotypes by African ancestry at HLA. Arch Neurol.

[25] Lucchinetti CF, Guo Y, Popescu BF, Fujihara K, Itoyama Y, Misu T (2014). The pathology of an autoimmune astrocytopathy: lessons learned from neuromyelitis optica. Brain Pathol.

[26] Ishizu T, Osoegawa M, Mei FJ (2005). Intrathecal activation of the IL-17/IL-8 axis in opticospinal multiple sclerosis. Brain.

[27] Matsushita T, Tateishi T, Isobe N (2013). Characteristic cerebrospinal fluid cytokine/chemokine profiles in neuromyelitis optica, relapsing remitting or primary progressive multiple sclerosis. PLoS ONE.

[28] Bennett JL, Lam C, Kalluri SR (2009). Intrathecal pathogenic anti-aquaporin-4 antibodies in early neuromyelitis optica. Ann Neurol.

[29] Bradl M, Misu T, Takahashi T (2009). Neuromyelitis optica: pathogenicity of patient immunoglobulin in vivo. Ann Neurol.

[30] Matsuya N, Komori M, Nomura K (2011). Increased T-cell immunity against aquaporin-4 and proteolipid protein in neuromyelitis optica. Int Immunol.

[31] Varrin-Doyer M, Spencer CM, Schulze-Topphoff U (2012). Aquaporin 4-specific T cells in neuromyelitis optica exhibit a Th17 bias and recognize *Clostridium* ABC transporter. Ann Neurol.

[32] Vaknin-Dembinsky A, Brill L, Kassis I (2012). T-cell reactivity against AQP4 in neuromyelitis optica. Neurology.

[33] Wolf PR, Ploegh HL (1995). How MHC class II molecules acquire peptide cargo: biosynthesis and trafficking through the endocytic pathway. Annu Rev Cell Dev Biol.

[34] Wucherpfennig KW, Strominger JL (1995). Molecular mimicry in T cell-mediated autoimmunity: viral peptides activate human T cell clones specific for myelin basic protein. Cell.

[35] Hemmer B, Gran B, Zhao Y (1999). Identification of candidate T-cell epitopes and molecular mimics in chronic Lyme disease. Nat Med.

[36] Markovic-Plese S, Hemmer B, Zhao Y, Simon R, Pinilla C, Martin R (2005). High level of cross-reactivity in influenza virus hemagglutinin-specific CD4+ T-cell response: implications for the initiation of autoimmune response in multiple sclerosis. J Neuroimmunol.

[37] Ley RE, Peterson DA, Gordon JI (2006). Ecological and evolutionary forces shaping microbial diversity in the human intestine. Cell.

[38] Cho I, Blaser MJ (2012). The human microbiome: at the interface of health and disease. Nat Rev Genet.

[39] Lynch SV, Pedersen O (2016). The human intestinal microbiome in health and disease. N Engl J Med.

[40] Rooks MG, Garrett WS (2016). Gut microbiota, metabolites and host immunity. Nat Rev Immunol.

[41] Levy M, Blacher E, Elinav E (2017). Microbiome, metabolites and host immunity. Curr Opin Microbiol.

[42] Ochoa-Reparaz J, Mielcarz DW, Begum-Haque S, Kasper LH (2011). Gut, bugs, and brain: role of commensal bacteria in the control of central nervous system disease. Ann Neurol.

[43] Miyake S, Kim S, Suda W (2015). Dysbiosis in the gut microbiota of patients with multiple sclerosis, with a striking depletion of species belonging to Clostridia XIVa and IV clusters. PLoS ONE.

[44] Tremlett H, Fadrosh DW, Faruqi AA (2016). Associations between the gut microbiota and host immune markers in pediatric multiple sclerosis and controls. BMC Neurol.

[45] Cekanaviciute E, Yoo BB, Runia TF, et al. Gut bacteria from multiple sclerosis patients modulate human T cells and exacerbate symptoms in mouse models. Proc Natl Acad Sci U S A 2017. 10.1073/pnas.1711235114 PMC5635915 28893978

[46] Berer K, Gerdes LA, Cekanaviciute E, et al. Gut microbiota from multiple sclerosis patients enables spontaneous autoimmune encephalomyelitis in mice. Proc Natl Acad Sci U S A 2017. 10.1073/pnas.1711233114 PMC5635914 28893994

[47] Mazmanian SK, Round JL, Kasper DL (2008). A microbial symbiosis factor prevents intestinal inflammatory disease. Nature.

[48] Ochoa-Reparaz J, Mielcarz DW, Ditrio LE (2010). Central nervous system demyelinating disease protection by the human commensal *Bacteroides fragilis* depends on polysaccharide A expression. J Immunol.

[49] Atarashi K, Tanoue T, Shima T (2011). Induction of colonic regulatory T cells by indigenous *Clostridium* species. Science.

[50] Atarashi K, Tanoue T, Oshima K (2013). Treg induction by a rationally selected mixture of Clostridia strains from the human microbiota. Nature.

[51] Ivanov II, Atarashi K, Manel N (2009). Induction of intestinal Th17 cells by segmented filamentous bacteria. Cell.

[52] Lee YK, Menezes JS, Umesaki Y, Mazmanian SK (2011). Proinflammatory T-cell responses to gut microbiota promote experimental autoimmune encephalomyelitis. Proc Natl Acad Sci U S A.

[53] Bettelli E, Carrier Y, Gao W (2006). Reciprocal developmental pathways for the generation of pathogenic effector TH17 and regulatory T cells. Nature.

[54] Cree BA, Spencer CM, Varrin-Doyer M, Baranzini SE, Zamvil SS (2016). Gut microbiome analysis in neuromyelitis optica reveals overabundance of *Clostridium perfringens*. Ann Neurol.

[55] Ohno H (2016). Intestinal M cells. J Biochem.

[56] Spencer J, Sollid LM (2016). The human intestinal B-cell response. Mucosal Immunol.

[57] Cong Y, Feng T, Fujihashi K, Schoeb TR, Elson CO (2009). A dominant, coordinated T regulatory cell-IgA response to the intestinal microbiota. Proc Natl Acad Sci U S A.

[58] Kawamoto S, Maruya M, Kato LM (2014). Foxp3(+) T cells regulate immunoglobulin a selection and facilitate diversification of bacterial species responsible for immune homeostasis. Immunity.

[59] Kubinak JL, Round JL (2016). Do antibodies select a healthy microbiota?. Nat Rev Immunol.

[60] Linhares UC, Schiavoni PB, Barros PO (2013). The ex vivo production of IL-6 and IL-21 by CD4+ T cells is directly associated with neurological disability in neuromyelitis optica patients. J Clin Immunol.

[61] Barros PO, Dias ASO, Kasahara TM (2017). Expansion of IL-6+ Th17-like cells expressing TLRs correlates with microbial translocation and neurological disabilities in NMOSD patients. J Neuroimmunol.

[62] Maslowski KM, Mackay CR (2011). Diet, gut microbiota and immune responses. Nat Immunol.

[63] Ivanov II, McKenzie BS, Zhou L (2006). The orphan nuclear receptor RORgammat directs the differentiation program of proinflammatory IL-17+ T helper cells. Cell.

[64] Honda K, Littman DR (2016). The microbiota in adaptive immune homeostasis and disease. Nature.

[65] Gupta RS, Gao B (2009). Phylogenomic analyses of clostridia and identification of novel protein signatures that are specific to the genus *Clostridium sensu stricto* (cluster I). Int J Syst Evol Microbiol.

[66] Wu C, Yosef N, Thalhamer T (2013). Induction of pathogenic TH17 cells by inducible salt-sensing kinase SGK1. Nature.

[67] Atarashi K, Tanoue T, Ando M (2015). Th17 cell induction by adhesion of microbes to intestinal epithelial cells. Cell.

[68] Sano T, Huang W, Hall JA (2015). An IL-23R/IL-22 circuit regulates epithelial serum amyloid A to Promote local effector Th17 responses. Cell.

[69] Sato DK, Callegaro D, Lana-Peixoto MA (2014). Distinction between MOG antibody-positive and AQP4 antibody-positive NMO spectrum disorders. Neurology.

[70] Kitley J, Waters P, Woodhall M (2014). Neuromyelitis optica spectrum disorders with aquaporin-4 and myelin-oligodendrocyte glycoprotein antibodies: a comparative study. JAMA Neurol.

[71] Hoftberger R, Sepulveda M, Armangue T (2015). Antibodies to MOG and AQP4 in adults with neuromyelitis optica and suspected limited forms of the disease. Mult Scler.

[72] Zamvil SS, Slavin AJ (2015). Does MOG Ig-positive AQP4-seronegative opticospinal inflammatory disease justify a diagnosis of NMO spectrum disorder?. Neurol Neuroimmunol Neuroinflamm.

[73] Carman RJ, Sayeed S, Li J (2008). Clostridium perfringens toxin genotypes in the feces of healthy North Americans. Anaerobe.

[74] Lindstrom M, Heikinheimo A, Lahti P, Korkeala H (2011). Novel insights into the epidemiology of Clostridium perfringens type A food poisoning. Food Microbiol.

[75] Rumah KR, Linden J, Fischetti VA, Vartanian T (2013). Isolation of *Clostridium perfringens* type B in an individual at first clinical presentation of multiple sclerosis provides clues for environmental triggers of the disease. PLoS ONE.

[76] Freedman JC, McClane BA, Uzal FA (2016). New insights into *Clostridium perfringens* epsilon toxin activation and action on the brain during enterotoxemia. Anaerobe.

[77] Dorca-Arevalo J, Soler-Jover A, Gibert M, Popoff MR, Martin-Satue M, Blasi J (2008). Binding of epsilon-toxin from *Clostridium perfringens* in the nervous system. Vet Microbiol.

[78] Jangi S, Gandhi R, Cox LM (2016). Alterations of the human gut microbiome in multiple sclerosis. Nat Commun.

[79] Tremlett H, Fadrosh DW, Faruqi AA (2016). Gut microbiota composition and relapse risk in pediatric MS: a pilot study. J Neurol Sci.

[80] Qi M, Nelson KE, Daugherty SC (2005). Novel molecular features of the fibrolytic intestinal bacterium Fibrobacter intestinalis not shared with *Fibrobacter succinogenes* as determined by suppressive subtractive hybridization. J Bacteriol.

[81] Jones MV, Huang H, Calabresi PA, Levy M (2015). Pathogenic aquaporin-4 reactive T cells are sufficient to induce mouse model of neuromyelitis optica. Acta Neuropathol Commun.

[82] Sagan SA, Winger RC, Cruz-Herranz A (2016). Tolerance checkpoint bypass permits emergence of pathogenic T cells to neuromyelitis optica autoantigen aquaporin-4. Proc Natl Acad Sci U S A.

[83] Vogel AL, Knier B, Lammens K (2017). Deletional tolerance prevents AQP4-directed autoimmunity in mice. Eur J Immunol.

[84] Sagan SA, Cruz-Herranz A, Spencer CM, et al. Induction of paralysis and visual system injury in mice by T cells specific for neuromyelitis optica autoantigen aquaporin-4. J Vis Exp 2017(126). 10.3791/56185 PMC5614352 28872108

[85] van Nood E, Vrieze A, Nieuwdorp M (2013). Duodenal infusion of donor feces for recurrent *Clostridium difficile*. N Engl J Med.

